# Dexketoprofen Time‐Dependent Administration After Third Molar Extraction: A Pilot Randomized Cross‐Over Controlled Trial

**DOI:** 10.1002/cre2.70163

**Published:** 2026-01-09

**Authors:** Fabián Pérez‐González, Mohammad Abusamak, Leire Virto Ruiz, Luis Miguel Sáez‐Alcaide, Haider Al‐Waeli, Faleh Tamimi, Jesus Torres García‐Denche

**Affiliations:** ^1^ Faculty of Dentistry, Department of Dental Clinical Specialties University Complutense of Madrid Madrid Spain; ^2^ Faculty of Dentistry, Dalhousie Halifax Nova Scotia Canada; ^3^ Department of Anatomy and Embriology, Faculty of Optics and Optometry University Complutense of Madrid Madrid Spain; ^4^ College of Dental Medicine Qatar University Doha Qatar

**Keywords:** chronotherapy, cytokines, NSAIDs, postoperative complications, third molar extraction

## Abstract

**Objective:**

To assess how chronotherapy influences recovery after bilateral third molar extraction when Dexketoprofen is administered as an anti‐inflammatory agent.

**Material and Methods:**

A randomized cross‐over controlled trial with 10 patients with bilateral impacted third molar extraction was enrolled. For 7 days after the surgery, the chronotherapy group was prescribed one dose of Dexketoprofen (25 mg) limited to daytime, while the control group were given two doses per day (every 12 h). Pain intensity was recorded at baseline, 24, 48, and 72 h, while facial swelling parameters were evaluated at baseline, 24, 72, and 168 h (Day 7) postoperatively. Also, the inflammatory profile was analyzed for each patient by blood samples at baseline, 72 and 168 h. The concentrations of IL‐1β, IL‐1a, IL‐2, IL‐4, IL‐6, IL‐7, IL‐10, and IL‐13 were measured using high‐sensitivity multiplex map human immunoassays.

**Results:**

No significant differences in postoperative VAS pain, swelling, and mouth opening (trismus) measures were observed between the two groups. However, the chronotherapy group had reduced overall postoperative complications compared to the control group. Regarding postoperative recovery, pain intensity scores in the chronotherapy group at 24, 48, and 72 h indicated a faster recovery than the control group. Blood samples analysis showed no statistical differences between the different inflammatory markers studied at 72 h or Day 7 in both groups.

**Conclusions:**

Daytime administration of NSAIDs might be sufficient to manage postoperative pain after third molar extraction, and complications were similar between both groups.

**Clinical Trial Registration:** clinicaltrials. gov, Identifier database: NCT05176158.

## 1 Introduction

The surgical removal of third molars, commonly known as wisdom teeth, is one of the most frequently performed oral surgeries in dentistry. In the United States alone, over 10 million wisdom teeth are extracted from around 5 million individuals each year, costing approximately 3 billion dollars (Friedman 2007). This procedure can lead to various postoperative complications, including pain, swelling, trismus, and bleeding (Chen et al. 2021). Additionally, these surgeries are often accompanied with significant anxiety, increased morbidity, and discomfort: the preoperative status of the patient may badly influence the postoperative patient's quality of life, particularly their ability to engage in everyday activities (Berge 1997; Landucci et al. 2016; Sancho‐Puchades et al. 2012; Monteiro et al. 2022; Parrini et al. 2012; Leonan‐Silva et al. 2025).

For this reason, to improve the quality of life of patients and to achieve a complete recovery, pain relief is a crucial step in the treatment of patients undergoing surgical interventions (Menhinick et al. 2004). Currently, acetaminophen, opioids, and nonsteroidal anti‐inflammatory drugs (NSAIDs) are prescribed to treat postoperative pain. However, acetaminophen is ineffective in managing severe pain, whereas NSAIDs may delay healing, and opioids can cause addiction and constipation (Mathiesen et al. 2014). Thus far, NSAIDs after surgeries are safe and routinely prescribed by surgeons; however, these drugs might slow postoperative pain and functional recovery (Moore et al. 2006).

In human, all vital physiological processes including pain perception, inflammation, bone healing process and drug absorption, distribution, metabolism, and elimination (ADME) are robustly regulated by the circadian rhythm (Abusamak et al. 2023, 2025). For instance, inflammatory pain perception tends to peak during the day, whereas neuropathic pain peaks at night (Moore et al. 2006; Abusamak et al. 2023). Additionally, postoperative plaque accumulation, food retention, and inflammation may also contribute to the intensity of postoperative pain (Camps‐Font et al. 2024; Falci et al. 2022; Parrini et al. 2023). NSAIDs are more effective in pain control when consumed during the day than at night due to circadian variation in ADME processes (Labrecque et al. 1995). Further, circadian clock core genes such as BMAL, CRY, and PER (1/2), which play an important role in bone formation and resorption, have been identified in bone tissue (Iimura et al. 2012; Dudek and Meng 2014). Based on these observations, chronotherapy is a promising field that is based on the knowledge of the biological clock during postsurgical healing processes and aims to improve efficacy and/or reduce side effects of drugs used in pain and inflammatory management. Recently, Al‐Waeli et al. 2020 investigated NSAIDs chronotherapy in a fracture model in mice (Al‐Waeli et al. 2020). They reported that restricting NSAIDs to daytime improved postoperative recovery (Al‐Waeli et al. 2020). Building on these preclinical findings, our research group evaluated the effect of NSAIDs chronotherapy on postoperative recovery after third molar extraction in two RCTs designs (Tamimi et al. 2022; Pérez‐González et al. 2023). We showed that postoperative pain follows a circadian rhythm and could be managed with daytime NSAIDs alone, while nighttime NSAIDs might not be necessary for pain control (Tamimi et al. 2022; Pérez‐González et al. 2023).

Moreover, this present RCT aimed to evaluate the effect of chronotherapy when Dexketoprofen is used as an anti‐inflammatory on postoperative recovery following bilateral third molar extraction surgery, utilizing an optimized study design. First, Dexketoprofen (25 mg) is typically prescribed twice daily (every 12 h), making it a more convenient regimen compared to ibuprofen (400 mg). Second, the follow‐up period for postoperative swelling was extended compared to our previous trials (3 days vs. 7 days), allowing for a more thorough assessment of recovery. The research question was: Among patients with bilateral impacted mandibular third molars indicated for extraction, does daytime administration of Dexketoprofen (one dose per day) improve postoperative recovery compared to the standard dosage regimen (two doses per day)?

## 2 Methods

### 2.1 Study Design

This study was designed as a pilot, cross‐over randomized, double‐blinded, placebo‐controlled, trial, conducted following the CONSORT 2010 statement (randomized crossover trials) (Dwan et al. 2019) and was carried out at the Oral Surgery Department of the Faculty of Dentistry at the Complutense University of Madrid (UCM) between March 2021 and December 2021. The study was evaluated and approved by the Research Ethics Committee at the San Carlos Clinical Hospital of Madrid, Spain (Trial registration code CEIC 19/216‐R_M_BNI.) and the *Agencia Española del Medicamento y Productos Sanitarios* (AEMPS: *Spanish Agency of Drugs and Sanitary Products*, EUDRACT number 2019–000,736) in April 2019. The study was registered at the Clinical Trials database with the following protocol number: NTC05176158. Patients were randomly assigned to the chronotherapy group in Period 1, followed by the control group in Period 2, or the control group in Period 1 followed by the chronotherapy group in Period 2. Each period was separated by at least 4‐week washout.

### 2.2 Population

Patients who attended the Postgraduate Program in Oral Surgery and Implant Dentistry at Complutense University (Madrid, Spain) for bilateral extraction of the lower third molar were recruited. The screening examination was performed by a 3rd‐year resident in the Oral Surgery and Implant Dentistry program (FPG) and included a medical and dental questionnaire and a standardized panoramic radiograph made at the Dental Radiology Service, Faculty of Dentistry, Complutense University of Madrid (CS 9300, Carestream Dental, Atlanta, GA, USA). Patients agreed to participate in the study by signing a written informed consent, in full accordance with the ethical principles of the Declaration of Helsinki on experimentation involving human.

### 2.3 Inclusion and Exclusion Criteria

The study included adults scheduled at the oral and maxillofacial surgery dental clinics for surgical extraction of third molars, restricted to bilateral lower third molars that are bony impacted to standardize the clinical cases. There is no significant difference between right and left impacted third molars in terms of mucosal coverage, bony coverage, and position, thus eliminating any effect due to side differences in each patient (Akarslan and Kocabay 2009). Patients were included if they (i) presented with a bilateral impacted lower third molars indicated for extraction (ii) aged between 18 and 35 years old, (iii) healthy according to the American Society of Anesthesiologists (ASA I–II) classification (patients should not have an active infection, trismus, hyperthermia, or swelling before surgery and adequate oral hygiene must be maintained), (iv) have an adequate understanding of written and spoken English or Spanish, and (v) be capable of signing an informed consent form and filling study questionnaires, and (v) agree to fully adhere to study instructions. The upper age limit was set to minimize age‐associated complications following third molar extraction as well as minimizing the risk of undiagnosed conditions (Lyons et al. 1980). Patients were not eligible for the study if they (i) had a history of systemic diseases (e.g., diabetes mellitus, hypertension, gastric ulcer), (ii) had a severe/serious illness that requires frequent hospitalization, (iii) were pregnant or breastfeeding, (iv) were allergic to anti‐inflammatory drugs, (v) have impaired cognitive or motor function, or (vi) were unable to return for evaluations/study recalls.

### 2.4 Sample Size Estimation

The sample size was set to provide minimum statistical power to estimate the effect of Dexketoprofen chronotherapy on postoperative pain intensity and facial swelling over time. Therefore, to attain 80% power for a two‐sided comparison of the two periods with estimated attrition of 10%, a sample size of 10 was required. The sample size was based on (i) treatment variability (SD) of primary outcome (VAS) was set to 1.0 point (Giorgetti et al. 2018), (ii) ability to detect a minimally clinically significant reduction of VAS pain mean difference of 1.5 points between treatment periods (Gallagher et al. 2001), and (iii) type I error rate of 0.05 (*p* < 0.05).

### 2.5 Randomization and Blinding

In the interest of minimizing bias, one clinician (FPG) performed the surgical procedure at 9:00 am for all the participants, and both the clinician and patients were blinded to treatment group allocation. The randomization was made by an external investigator (MA) who had no relation with the surgical procedure. For this purpose, after checking eligibility criteria, the investigator generated a list of randomization sequence using permuted‐block randomization (1:1) via the website www.randomization.com assigning patients to chronotherapy or control groups. Another investigator (J.T.) distributed the postoperative medications in the appropriate sequence. Data analysis was carried out by an independent investigator (M.A.).

### 2.6 Groups

The patients were enrolled in both groups: control and experimental. In the control group, the patients were prescribed with Dexketoprofen 25 mg in the morning (8 a.m.) and at night (8 p.m.) for 7 days, while the chronotherapy group (experimental) received Dexketoprofen 25 mg in the morning (8 a.m.) and a placebo capsule at night (8 p.m.) for 7 days.

### 2.7 Intervention

For each surgery, one and a half carpule of 4% articaine with 1:100,000 adrenaline (Ultracaín, Normon SL, Madrid, Spain) were used to anaesthetize the inferior alveolar, lingual, and long buccal nerves. A full‐thickness mucoperiosteal envelope flap was raised in all cases. When necessary, bone removal and/or tooth sectioning were performed with a cooled, sterile saline‐irrigated surgical handpiece and a 701 surgical bur (drill). Sharp bony socket edges were smoothened, followed by curettage of the distal aspect of the second molar, and socket lavage with sterile saline solution. Flap closure was performed with 4/0 silk sutures (Aragó, Barcelona, Spain). Surgery time, surgery difficulty according to Parant scale (Janjua et al. 2013), and surgical complications were recorded. Patients were provided with written and verbal postoperative instructions and prescribed amoxicillin 750 mg every 8 h for 7 days. After at least 1 month, participants underwent surgical extraction of the contralateral lower third molar, and those who received a placebo following the previous surgery received two doses instead and vice versa. The postoperative medications were given to each patient blindly. Lower left impacted third molar (3.8) was surgically extracted in Period 1, while the lower right impacted third molar (4.8) was extracted in Period 2. All procedures were developed by the same surgeon, with 5 years' experience in oral surgery.

### 2.8 Outcomes Measures

Pain intensity was the primary outcome and recorded at baseline, 24, 48, and 72 h postoperatively using a segmented numeric version of the visual analog scale (VAS) (Rodriguez 2001). In addition, patients were instructed to record the time of maximum pain perceived and the day they stopped perceiving pain. As a secondary outcome, facial swelling parameters such as distance from Tragus to Pogonin (Tg‐Pg) (Amin and Laskin 1983) and trismus (i.e., interincisal distance) were evaluated at baseline, 24, 72, and 168 h postoperatively. For ethical reasons, all patients received rescue medication (650 mg acetaminophen) and were also recorded (day/time/number of pills).

Also, the inflammatory profile was analyzed for each patient from blood samples. A venous blood sample of 9 cc was drawn from the cubital fossa of each patient three times (once preoperatively, at 72 and 168 h postoperatively). The choice of timing was based on prior studies indicating that C‐Reactive Protein (CRP), swelling, and inflammatory cytokine levels, particularly IL‐1β and IL‐6, typically peak around 48–72 h post‐surgery (Kim et al. 2009; Chander et al. 2013). The samples were collected and analyzed in the Laboratory of the Dentistry Faculty in the Complutense University of Madrid by an independent analyst (LV). The concentrations of IL‐1β, IL‐1a, IL‐2, IL‐4, IL‐6, IL‐7, IL‐10, and IL‐13 were measured using high‐sensitivity multiplex map human immunoassays (Millipore, Cat. # HCYTA‐60K‐08, Billerica, MA, USA) using a Luminex‐200 System Unit with the XY platform (Luminex, Oosterhout, the Netherlands). Results were measured using the dedicated software (xPonent software; Luminex Corporation) and were expressed as picograms per milliliter.

### 2.9 Statistical Analysis

Distribution of population parameters and clinical variables (mean and standard deviation) was obtained. After testing for normality, paired t tests were used. Data analyzed with R statistical program (4.2.3) by an independent investigator (MA). As for the blood test analysis, after testing for normality, nonparametric paired test (Wilcoxon Signed‐Rank test) for independent samples was used, comparing the control and the experimental group at 72 h and Day 7, with a Type I error rate of 0.05 (*p* < 0.05).

## 3 Results

**Figure 1:**
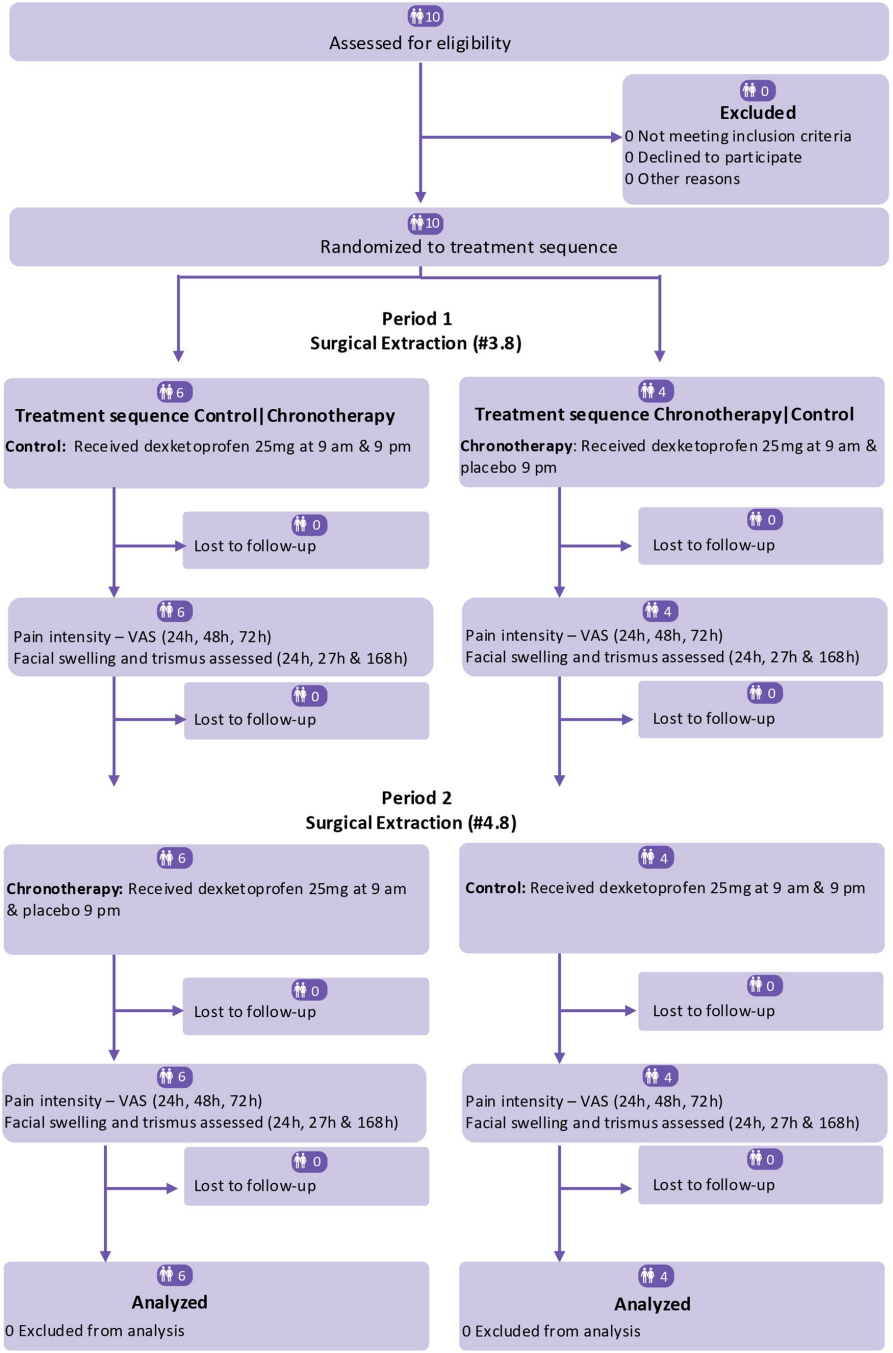
CONSORT flow diagram.

Ten patients (4 males and 6 females), aged 18–22 years (mean 20.3 ± 1.06), were enrolled (Figure 1). Tables 1 and 2, describe demographics and clinical variables for both periods and treatment sequences. Overall, demographics and clinical variables were balanced and homogenously distributed except swelling baseline measures in both periods one and two (Table 1). However, treatment sequence two had three females and one male included, while treatment sequence one gender distribution was even (Table 2).

**Table 1:** Demographic and clinical variables per study period.

Demographic variables		
Age [mean (SD)]	20.3 (1.06)	
Sex	4 Males; 6 Females	
Race	4 Asia; 6 Cauca	

	Period 1	
Clinical variables	Control (*n* = 6)	Chronotherapy (*n* = 4)
Extracted tooth	3.8	
Impaction type		
Full bone impaction	0	1
Partial bone impaction	6	3
Impaction position		
Mesial	3	3
Vertical	1	1
Horizontal	2	0
Surgical procedure*		
II	1	4
III	4	0
IV	1	0
Surgery duration [mean (SD)], min	13.06 (2.99)	10.81 (4.39)
**Baseline measurements**		
VAS Pain Scores	0	0
Swelling [mean (SD)], mm*	156.50 (8.22)	142.25 (5.38)
Mouth opening [mean (SD)], mm	52.17 (5.04)	46 (5.48)
Surgical complications	No	

	Period 2	
Clinical variables	Chronotherapy (*n* = 6)	Control (*n* = 4)
Extracted tooth	4.8	
Impaction type		
Full bone impaction	0	1
Partial bone impaction	6	3
Impaction position		
Mesial	3	2
Distal	1	0
Horizontal	2	2
Surgical procedure		
II	2	1
III	2	2
IV	2	1
Surgery duration [Mean (SD)], mins	14.89 (7.90)	12.35 (2.88)
**Baseline measurements**		
VAS Pain Scores	0	0
Swelling [Mean (SD)], mm*	155.00 (7.46)	144.50 (4.04)
Mouth opening [Mean (SD)], mm	49.67 (5.09)	44.25 (4.57)
Surgical complications		
No	5	4
Yes (Hemorrhage)	1	0

* Unbalanced distribution. All other demographic and clinical variables were equally distributed (*p* > 0.05).

**Table 2:** Demographic and clinical variables per treatment sequence.

Demographic variables			
Age [mean (SD)]	20.3 (1.06)		
Sex	4 Males; 6 Females		
Race	4 Asia; 6 Cauca		

	Sequence control|Chronotherapy		
Clinical variables	**Control (*n*** = **6)**	**Chronotherapy (*n*** = **6)**	
Extracted tooth	3.8	4.8	
Impaction type			
Partial bone impaction	6	6	
Impaction position			
Mesial	3	3	
Distal	0	1	
Vertical	1	0	
Horizontal	2	2	
Surgical procedure			
II	1	2	
III	4	2	
IV	1	2	
Surgery duration [Mean (SD)], min	13.06 (2.99)	14.89 (7.90)	
Baseline measurements			
VAS Pain Scores	0	0	
Swelling [Mean (SD)], mm	156.50 (8.22)	155.00 (7.46)	
Mouth opening [Mean (SD)], mm	52.17 (5.04)	49.67 (5.09)	
Surgical complications	No		

	Sequence chronotherapy|Control		
Clinical variables	Chronotherapy (*n* = 4)	Control (*n* = 4)	
Extracted tooth	3.8	4.8	
Impaction type			
Full bone impaction	1	1	
Partial bone impaction	3	3	
Impaction position			
Mesial	3	2	
Vertical	1	0	
Horizontal	0	2	
Surgical procedure			
II	4	1	
III	0	2	
IV	0	1	
Surgery duration [Mean (SD)] ‐ mins	10.81 (4.39)	12.35 (2.88)	
**Baseline measurements**			
VAS Pain Scores	0	0	
Swelling [mean (SD)], mm	142.25 (5.38)	144.50 (4.04)	
Mouth opening [Mean (SD)], mm	46.00 (5.48)	44.25 (4.57)	
Surgical complications			
No	5	4	
Yes (Hemorrhage)	1	0	

**Figure 2:**
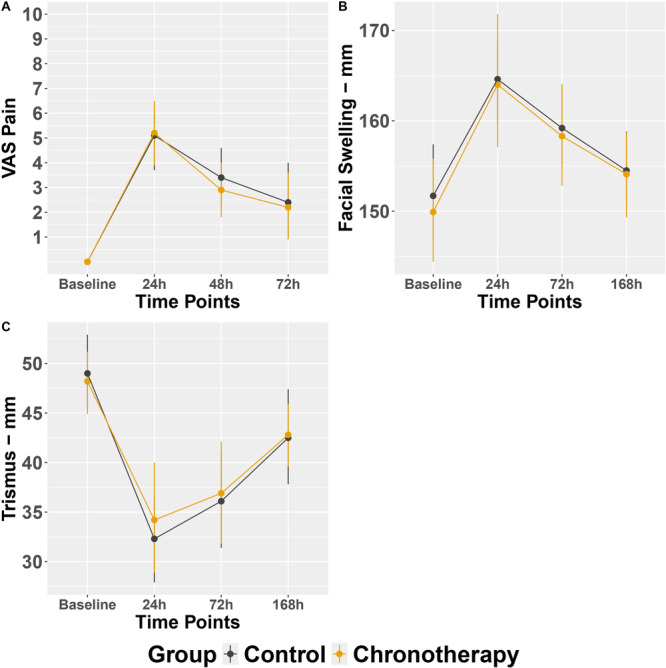
Postoperative outcomes measures: (A) postoperative pain; (B) postoperative facial swelling; and (C) postoperative trismus.

While there were no significant differences in postoperative VAS pain, swelling and mouth opening (trismus) measures between experimental groups, chronotherapy group had reduced overall postoperative complications compared to control group (Tables 3, 4, 5, Figure 2, *p* > 0.05). Similarly, both groups did not differ in rescue medication consumption. However, on average, the highest perceived pain was reported at 19:00 h and 16:30 h for the control and chronotherapy groups, respectively (*p* < 0.05).

**Table 3:** Postoperative measurements between experimental groups.

	Control, *n* = 10	Chronotherapy, *n* = 10	*p* *
**Primary outcome**			
VAS Pain 24 h [mean (SD)]	5.10 (2.18)	5.20 (2.30)	> 0.05
VAS Pain 48 h [mean (SD)]	3.40 (1.96)	2.90 (1.91)	> 0.05
VAS Pain 72 h [mean (SD)]	2.40 (2.67)	2.20 (2.44)	> 0.05
No pain reported [mean (SD)], days	4.11 (1.27)	4.40 (1.58)	> 0.05
Time of max. pain [mean (SD)], h	18:46 (1:38)	16:39 (2:07)	**< 0.05**
**Secondary outcome**			
Facial swelling, %			
24 h [mean (SD)]	8.67 (5.27)	9.57 (8.06)	> 0.05
72 h [mean (SD)]	5.07 (3.41)	5.69 (4.59)	> 0.05
7 days [mean (SD)]	1.93 (2.27)	2.92 (4.17)	> 0.05
**Mouth opening**			
Trismus, %			
24 h [mean (SD)]	57.66 (32.65)	52.77 (53.11)	> 0.05
72 h [mean (SD)]	40.94 (32.08)	37.01 (36.72)	> 0.05
7 days [mean (SD)]	18.13 (21.38)	14.17 (19.60)	> 0.05
Total RM consumed [mean (SD)]	2.60 (3.06)	3.40 (3.69)	> 0.05

* Paired *t*‐test.

**Table 4:** Postoperative measurements between experimental groups per period.

	Period 1			Period 2		
	Control (*n* = 6)	Chronotherapy (*n* = 4)	*p* *	Chronotherapy (*n* = 6)	Control (*n* = 4)	*p* *
**Primary outcome**						
VAS Pain 24 h [mean (SD)]	6.17 (1.33)	4.25 (2.22)	> 0.05	5.83 (2.32)	3.50 (2.38)	> 0.05
VAS Pain 48 h [mean (SD)]	3.83 (1.72)	2.25 (2.22)	> 0.05	3.33 (1.75)	2.75 (2.36)	> 0.05
VAS Pain 72 h [mean (SD)]	2.83 (3.25)	1.00 (1.41)	> 0.05	3.00 (2.76)	1.75 (1.71)	> 0.05
No pain reported [mean (SD)], days	4.17 (1.17)	4.25 (1.71)	> 0.05	4.50 (1.64)	4.00 (1.73)	> 0.05
Time of max. pain [mean (SD)], h	19:32 (1:30)	16:30 h (2:12)	**< 0.05**	16.45 (2.16)	17:40 (1:32)	> 0.05
**Secondary outcome**						
Facial swelling, % (postop – preop)						
24 h [mean (SD)]	7.18 (4.61)	13.35 (8.41)	> 0.05	7.05 (7.44)	10.92 (6.05)	> 0.05
72 h [mean (SD)]	3.69 (3.26)	6.94 (5.59)	> 0.05	4.85 (4.13)	7.12 (2.79)	> 0.05
7 days [mean (SD)]	0.76 (0.78)	5.39 (5.92)	> 0.05	1.27 (1.47)	3.69 (2.76)	> 0.05
**Mouth opening**						
Trismus, % (preop – postop)						
24 h [mean (SD)]	51.73 (32.15)	61.20 (70.52)	> 0.05	47.15 (44.71)	66.56 (36.06)	> 0.05
72 h [mean (SD)]	41.17 (36.32)	46.17 (47.22)	> 0.05	30.90 (31.27)	40.61 (29.81)	> 0.05
7 days [mean (SD)]	14.88 (16.67)	19.07 (29.18)	> 0.05	10.90 (12.18)	23.02 (29.25)	> 0.05
Total RM consumed [mean (SD)]	3.67 (3.50)	1.25 (0.96)	> 0.05	4.83 (4.22)	1.00 (1.41)	> 0.05

* One‐way ANOVA.

**Table 5:** Postoperative measurements between experimental groups per sequence.

	Sequence control|Chronotherapy			Sequence chronothearpy|Control		
	Control (*n* = 6)	Chronotherapy (*n* = 6)	*p* *	Chronotherapy (*n* = 4)	Control (*n* = 6)	*p* *
**Primary outcome**						
VAS Pain 24 h [mean (SD)]	6.17 (1.33)	5.83 (2.32)	> 0.05	4.25 (2.22)	3.50 (2.38)	> 0.05
VAS Pain 48 h [mean (SD)]	3.83 (1.72)	3.33 (1.75)	> 0.05	2.25 (2.22)	2.75 (2.36)	> 0.05
VAS Pain 72 h [mean (SD)]	2.83 (3.25)	3.00 (2.76)	> 0.05	1.00 (1.41)	1.75 (1.71)	> 0.05
No pain reported [mean (SD)], days	4.17 (1.17)	4.25 (1.71)	> 0.05	4.25 (1.71)	4.00 (1.73)	> 0.05
Time of max. pain [mean (SD)], h	19:32 (1:30)	16:45 h (2:16)	**< 0.05**	16.30 (2.16)	17:40 (1:32)	> 0.05
**Secondary outcome**						
Facial swelling, %						
24 h [mean (SD)]	7.18 (4.61)	7.05 (7.44)	> 0.05	13.35 (8.41)	10.92 (6.05)	> 0.05
72 h [mean (SD)]	3.69 (3.26)	4.85 (4.13)	> 0.05	6.94 (5.59)	7.12 (2.79)	> 0.05
7 days [mean (SD)]	0.76 (0.78)	1.27 (1.47)	> 0.05	5.39 (5.92)	3.69 (2.76)	> 0.05
**Mouth opening**						
Trismus, %						
24 h [mean (SD)]	51.73 (32.15)	47.15 (44.71)	> 0.05	61.20 (70.52)	66.56 (36.06)	> 0.05
72 h [mean (SD)]	41.17 (36.32)	30.90 (31.27)	> 0.05	46.17 (47.22)	40.61 (29.81)	> 0.05
7 days [mean (SD)]	14.88 (16.67)	10.90 (12.18)	> 0.05	19.07 (29.18)	23.02 (29.25)	> 0.05
Total RM consumed [mean (SD)]	3.67 (3.50)	4.83 (4.22)	> 0.05	1.25 (0.96	1.00 (1.41)	> 0.05

* Paired *t*‐test.

**Figure 3:**
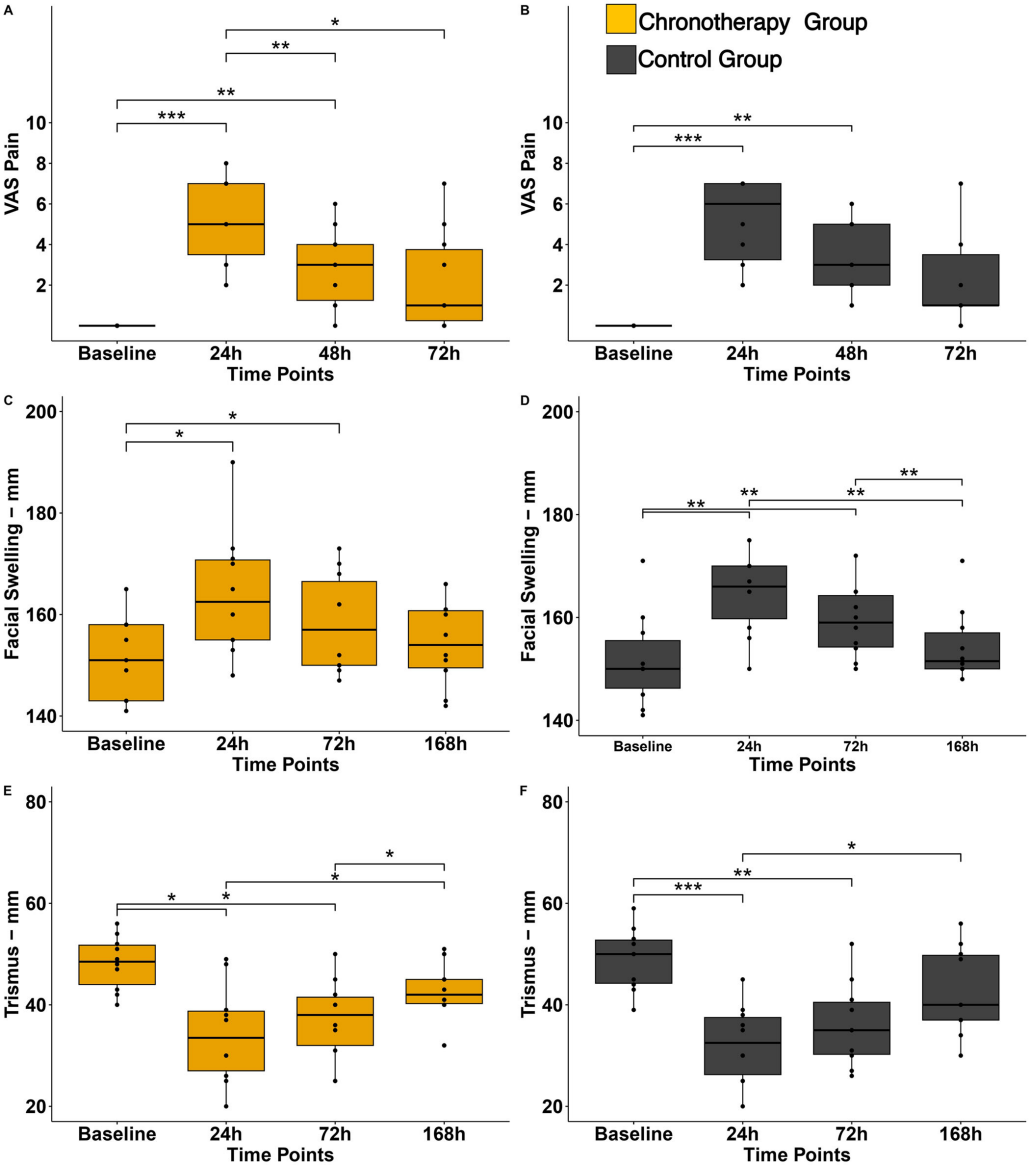
Postoperative recovery: (A) postoperative pain recovery for chronotherapy group; (B) postoperative pain recovery for control group; (C) postoperative facial swelling recovery for chronotherapy group; (D) postoperative facial swelling recovery for control group; (E) postoperative trismus recovery for chronotherapy group; and (F) postoperative trismus recovery for control group.

Moreover, we looked at each group separately in terms of postoperative recovery (VAS pain, swelling, and trismus). The chronotherapy group exhibited a faster recovery pattern over time. Specifically, VAS pain scores in the chronotherapy group significantly declined between 24, 48, and 72 h (Figure 3A), whereas the control group showed no significant changes across time points (Figure 3B). Additionally, the time of maximum pain perception occurred earlier in the chronotherapy group (mean 16:39 h) than in the control group (mean 18:46 h), a statistically significant difference (*p* < 0.05). These findings suggest a more rapid pain resolution trend in the chronotherapy group. Finally, VAS pain scores at 72 h reported for both groups were not significantly different from baseline scores (Figure 3A,B).

As to postoperative swelling recovery, both swelling measurements at 24 and 72 h were significantly different from swelling measurements at Day 7 in the control group (Figure 3C). Conversely, the chronotherapy group had no significant measurement differences between 24 h, 72 h and Day 7 (Figure 3D). Like VAS pain scores, last swelling measurements (i.e., Day 7) in both groups were close to their respective baseline measurements (Figure 3C,D). Lastly, the control group exhibited significant differences in mouth opening measurements between 24 h and Day 7 time points, whereas the chronotherapy group showed significant differences between 24 h – Day 7 and 72 h – Day 7 time points showing faster recovery (Figure 3E,F). This specific pattern was also found in VAS pain scores of chronotherapy group (Figure 3A).

**Figure 4:**
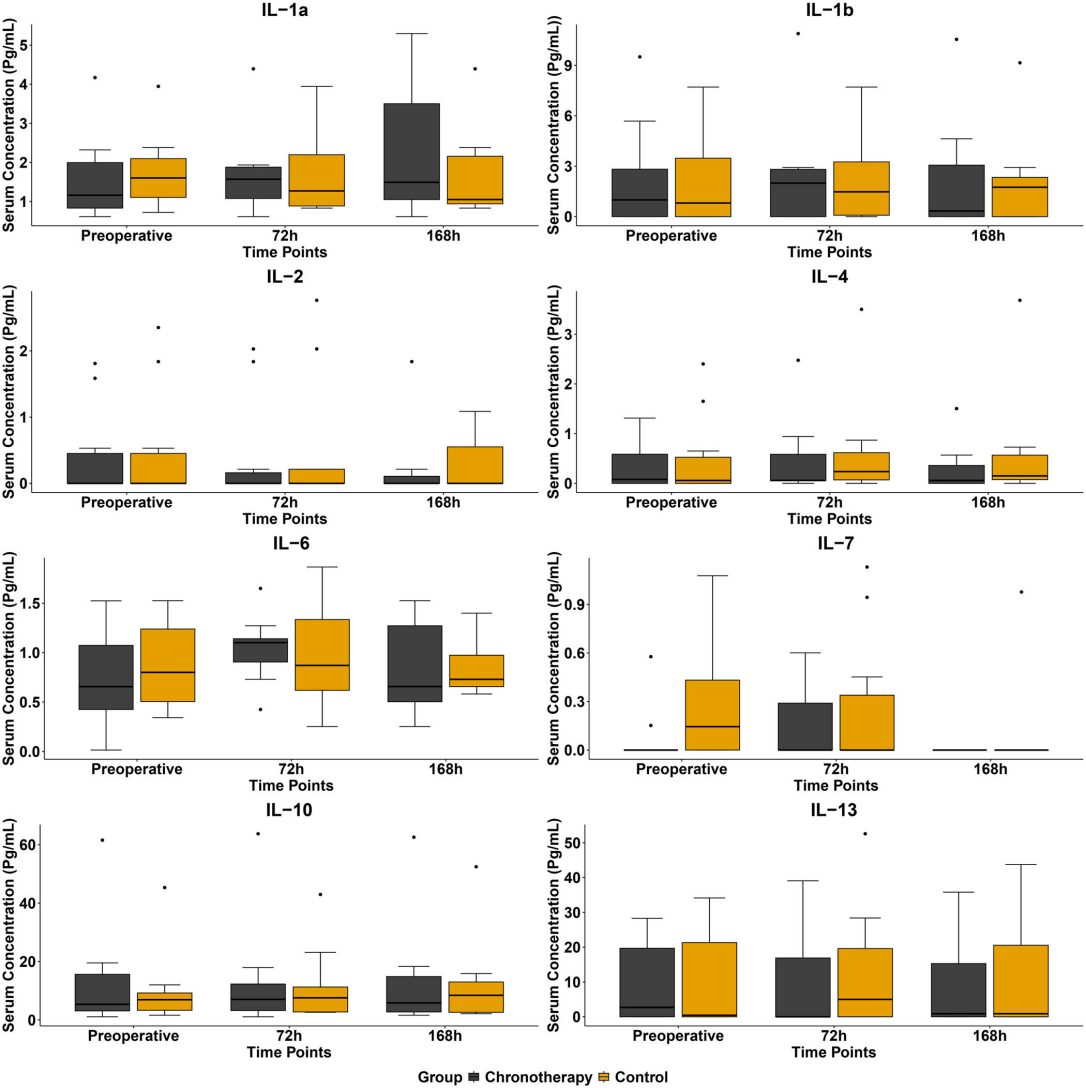
Pre‐ and postoperative interleukin serum concentration levels between experimental groups.

The results of the inflammatory profile showed that in the chronotherapy group, the concentration of the pro‐inflammatory interleukin IL‐1a increased slightly after surgery with respect to baseline levels, while the other pro‐inflammatory interleukins IL1‐β, IL‐2, and IL‐6 remained unchanged. As for the anti‐inflammatory interleukins, there was a slight increase in IL‐10 from baseline levels and a decrease in IL‐4 and IL‐13. However, no significant differences were observed in the concentration of pro‐ and anti‐inflammatory cytokines either with respect to baseline levels or between the chronotherapy and control groups at 72 and 168 h postoperatively (Figure 4).

## 4 Discussion

The aim of this RCT was to evaluate the effect of chronotherapy when Dexketoprofen is used as an anti‐inflammatory in postoperative third molar extraction surgery. For this purpose, a crossover study was designed, where in the experimental group only one daytime intake was prescribed while in the control group two intakes were prescribed, one in the morning and one in the evening (conventional dosage). Different postoperative clinical records were taken, such as pain intensity and facial swelling as well as several inflammatory concentration markers in serum.

Chronotherapy is a novel field that attempts to synchronize drug administration with circadian rhythms. It has been observed in different studies that its use improves therapeutic efficacy and reduces side effects (Poornachitra et al. 2023). In contrast to this philosophy, the current approach to achieve therapeutic drug levels is to increase drug dosage concentration or frequency. Chronotherapy is not yet included commonly in medical practice, despite having shown benefits in several medical treatments as inflammatory disorders, cardiovascular disease, asthma, or even different cancer treatments (Swanson et al. 2023).

It has been demonstrated that different chemical agents have been shown to be more effective and produce fewer toxic side effects when given at certain times of the day. For example, asthma symptoms present a circadian variation being worse and more prevalent at night; In cholesterol synthesis significant differences have been found in the effectiveness of lowering cholesterol receiving the treatment in the evening versus the morning; and in cancer treatment, chemotherapy and radiotherapy is more effective for patients in terms of increasing efficacy and decreasing unwanted side effects, depending on the time of the day (Butler et al. 2024). Chronotherapy is a rapidly growing field, and we will soon see more and more studies focusing even on ‘personalized chronotherapy’.

Recent investigation reported that the expression of the genes involved in the circadian clock pathway and circadian rhythm biological process was affected by NSAID chronotherapy (Al‐Waeli et al. 2020). Circadian variations in NSAID pharmacokinetics, cytokine release, and COX activity would imply the possibility of establishing a chronotherapeutic treatment that would maximize the effect of NSAIDs while reducing their postoperative side effect (Tamimi et al. 2022; Pérez‐González et al. 2023).

This RCT suggests that Dexketoprofen chronotherapy is a promising strategy. Although chronotherapy group had NSAIDs during daytime only and the control group had two doses (daytime and nighttime), postoperative recovery outcomes after third molar extraction were comparable. While there were no statistically significant differences in group mean VAS pain scores at each time point (24, 48, and 72 h), the pattern of recovery within the chronotherapy group showed a more pronounced and statistically significant decline in pain over time, as illustrated in Figure 3A,B. Specifically, VAS pain scores in the chronotherapy group significantly decreased from 24 h to 48 h and 72 h, whereas the control group showed no significant changes between time points, indicating a more gradual recovery. These findings, along with the trajectory of swelling and trismus in Figure 3C–F, support the interpretation that patients in the chronotherapy group experienced a faster overall recovery, despite the lack of significant differences at individual time points when comparing groups. These findings were also in accordance with our previous clinical trials demonstrating the potential of chronotherapy not only in managing postoperative pain with fewer pain pills but also developing the concept for targeted nighttime pill that will improve neuropathic pain control (Tamimi et al. 2022; Pérez‐González et al. 2023). Further, our study suggests that 7 days postoperative follow‐up to assess swelling and pain might not be long enough for all measures to return to baseline scores.

Circadian rhythm oscillation was found in several conditions related to pain; for example, neuropathic and temporomandibular joint pain became intolerable around 8 p.m., while patients with trigeminal neuralgia and fibromyalgia experienced a marked increase in pain in the morning (Yang et al. 2024). Yang et al. demonstrated that the administration of analgesics during the active period was superior to that administered during the resting period (Yang et al. 2024). Although the chronotherapy group was prescribed daytime Dexketoprofen only, we found no differences in terms of pain perception between groups; this suggests that the peak pain in patients undergoing oral surgery involving ostectomy occurs during the daytime phase and that a single intake during this phase is sufficient to control postoperative discomfort.

Growing evidence in the literature highlights the importance of time as a critical factor in medicine for developing effective treatments across various pathological conditions. Notably, advancements in chronotherapy are largely dependent on the development of circadian precision medicine, where treatments are aligned with the natural rhythms of target physiological processes. Accordingly, rhythmic fluctuations in drug absorption, distribution, metabolism, excretion, and the expression of drug targets have been demonstrated to significantly influence both pharmacokinetics and pharmacodynamics. It can be concluded that circadian rhythms play a pivotal role in the onset and progression of inflammatory diseases, with diurnal variations observed in both the expression of disease symptoms and the associated inflammatory processes (Xu et al. 2021). For example, the activity of macrophages, leukocyte recruitment, and pro‐inflammatory mediators such as interleukin‐1β (IL‐1β) or interleukin‐6 (IL‐6), tend to increase at the onset of daily activity. During this period, the levels of Toll‐Like Receptors (TLR9 and TLR4) also rise, promoting the upregulation of CCL2, CXCL1, and CCL5, which further enhance leukocyte recruitment and may contribute to tissue damage at sites of injury. Conversely, anti‐inflammatory mediators and factors associated with growth and angiogenesis reach their highest levels during the resting phase (Al‐Waeli et al. 2020). In that sense, NSAID intake during the active phase (diurnal) could modulate the synthesis and release of cytokines by the cyclooxygenase inhibition, causing a decrease in the pro‐inflammatory cytokines (like IL‐1 β) and promoting the anti‐inflammatory cytokines (such as IL‐13 and IL‐14) (Al‐Waeli et al. 2020).

To analyze the inflammatory profile of patients, we performed an analysis of different pro‐inflammatory (IL‐1β, IL‐1a, IL‐2, IL‐6, and IL‐7) and anti‐inflammatory (IL‐4, IL‐10, and IL‐13) cytokines during the postoperative period. However, we did not observe significant differences between the two groups in the levels of pro‐inflammatory or anti‐inflammatory interleukins, indicating that a single morning intake does not compromise the efficacy of the treatment. Further, future studies might consider adding earlier timepoints (e.g., 6 h and 24 h) for blood inflammatory markers analysis to provide a more comprehensive characterization of the early postoperative inflammatory response.

The results of this study suggest that administering the medication once daily in the morning, as done with the chronotherapy group, might be as effective as the twice‐daily dosing regimen used in the control group. This aligns with the understanding that circadian rhythms play a role in inflammatory processes, and targeting these rhythms with a morning dose may be sufficient to achieve therapeutic outcomes, potentially simplifying treatment regimens.

Compared with other analgesic and anti‐inflammatory drugs, ibuprofen doses above 400 mg achieve a high level of analgesic efficacy, but further increases provide minimal additional benefit. In contrast, a combination of paracetamol 1000 mg and codeine 60 mg offers comparable analgesia with a faster onset of action. Furthermore, dexketoprofen demonstrates greater analgesic potency and a longer duration of action than commonly used analgesics like ibuprofen or paracetamol (Xu et al. 2021).

Circadian rhythms are not the sole contributors to variations in inflammation and pain‐related behaviors; accumulating evidence also indicates that bone and cartilage metabolism follow distinct diurnal patterns, with most bone metabolic markers exhibiting rhythmic fluctuations characterized by nocturnal peaks and diurnal troughs (Ze et al. 2025).

Nevertheless, this RCT has several limitations. First, there were unequal numbers of males and females between treatment sequences. Second, this pilot study had a small sample size. However, this RCT was conducted following a cross‐over design, empowering the results. Therefore, future multicentric RCTs with larger populations and longer follow‐ups (e.g., 14 days or more) are needed to confirm our findings. Moreover, men's circadian cycles are longer than women's, indicating that men and women have different biological clocks (Lévi et al. 2024), and the response to pain is different among sex and that was not considered (Abusamak et al. 2023, 2025). Consequently, further investigations should implement circadian‐based protocols in future studies to improve the reliability and internal validity, considering also the sex or group of age. Next, our initial sample size calculation was based on an anticipated standard deviation of 1.0 for the primary outcome (VAS pain scores), as informed by previous literature. However, the standard deviations observed in our study were higher (ranging from 1.9 to 2.6), which likely reduced the actual statistical power. As a result, the study may have been underpowered to detect a clinically meaningful difference of 1.5 points in VAS pain scores. Future studies should consider a larger sample size to account for this variability and better assess the potential benefits of dexketoprofen chronotherapy. Finally, this pilot study only recorded one facial swelling landmark (Tragus to Pogonin – DHC), and future studies might consider three landmarks for facial swelling measures as described in the Laskin method.

## 5 Conclusion

In conclusion, notwithstanding the limitations of the current study, daytime administration of NSAIDs may be as effective as twice‐daily dosing in young adults to manage postoperative pain after third molar extraction. However, futures multicentric RCTs with larger population are needed to confirm our findings. Even though the chronotherapy group was only receiving half of the dosage received by the control group, postoperative complications were similar between both groups.

## Author Contributions

Fabián Pérez‐González and Mohammad Abusamak conceived the research idea, Mohammad Abusamak developed the methodology, and Fabián Pérez‐González performed the treatment. Fabián Pérez‐González and Mohammad Abusamak interpreted the data and drafted, revised, formatted, and edited the manuscript. Leire Virto, Jesus Torres, Luis Miguel Sáez, Haider Al‐Waeli, and Faleh Tamimi co‐conceived the research idea and supervised the methodology. All authors gave their approval to the final version of this manuscript.

## Ethics Statement

The study was evaluated and approved by the Research Ethics Commitee at the San Carlos Clinical Hospital of Madrid, Spain (Trial registration code CEIC 19/216‐R_M_BNI) and the *Agencia Española del Medicamento y Productos Sanitarios* (AEMPS: *Spanish Agency of Drugs and Sanitary Products*, EUDRACT number 2019–000,736).

## Consent

Informed consent was given for each patient, and it was written in the Material and Methods section.

## Conflicts of Interest

The authors declare no conflicts of interest.

## Data Availability

The data that support the findings of this study are available from the corresponding author upon reasonable request.
